# IL-10 producing regulatory B cells are decreased in blood from smokers and COPD patients

**DOI:** 10.1186/s12931-022-02208-1

**Published:** 2022-10-17

**Authors:** Merel Jacobs, Sven Verschraegen, Bihiyga Salhi, Jasper Anckaert, Pieter Mestdagh, Guy G. Brusselle, Ken R. Bracke

**Affiliations:** 1 Laboratory for Translational Research in Obstructive Pulmonary Diseases, Department of Respiratory Medicine, Ghent University Hospital, C. Heymanslaan 10, 9000 Ghent, Belgium; 2 Department of Respiratory Medicine, Ghent University Hospital, Ghent, Belgium; 3 Center for Medical Genetics, Department of Biomolecular Medicine, Ghent University, Ghent, Belgium; 4 Department of Epidemiology, Erasmus Medical Center, Rotterdam, The Netherlands; 5 Department of Respiratory Diseases, Erasmus Medical Center, Rotterdam, The Netherlands; 6 OncoRNALab, Cancer Research Institute Ghent (CRIG), Ghent University, Ghent, Belgium

**Keywords:** Regulatory B cells, COPD (chronic obstructive pulmonary disease), Cigarette smoke, IL-10 (interleukin-10)

## Abstract

**Background:**

Two opposing B cell subsets have been defined based on their cytokine profile: IL-6 producing effector B cells (B-effs) versus IL-10 producing regulatory B cells (B-regs) that respectively positively or negatively regulate immune responses. B-regs are decreased and/or impaired in many autoimmune diseases and inflammatory conditions. Since there is increasing evidence that links B cells and B cell-rich lymphoid follicles to the pathogenesis of COPD, the aim of this study was to investigate the presence and function of B-regs in COPD.

**Methods:**

First, presence of IL-10 producing regulatory B cells in human lung tissue was determined by immunohistochemistry. Secondly, quantification of IL-10 + B-regs and IL-6 + B-effs in peripheral blood mononuclear cells (PBMCs) from healthy controls, smokers without airflow limitation, and COPD patients (GOLD stage I-IV) was performed by flow cytometry. Thirdly, we exposed blood-derived B cells from COPD patients in vitro to cigarette smoke extract (CSE) and quantified IL-10 + B-regs and IL-6 + B-effs. Furthermore, we aimed at restoring the perturbed IL10 production by blocking BAFF. Fourthly, we determined mRNA expression of transcription factors involved in IL-10 production in FACS sorted memory- and naive B cells upon exposure to medium or CSE.

**Results:**

The presence of IL-10 producing regulatory B cells in parenchyma and lymphoid follicles in lungs was confirmed by immunohistochemistry. The percentage of IL-10 + B-regs was significantly decreased in blood-derived memory B cell subsets from smokers without airflow limitation and patients with COPD, compared to never smokers. Furthermore, the capacity of B cells to produce IL-10 was reduced upon in vitro exposure to CSE and this could not be restored by BAFF-blockade. Finally, upon CSE exposure, mRNA levels of the transcription factors IRF4 and HIF-1α, were decreased in memory B cells.

**Conclusion:**

Decreased numbers and impaired function of B-regs in smokers and patients with COPD might contribute to the initiation and progression of the disease.

**Supplementary Information:**

The online version contains supplementary material available at 10.1186/s12931-022-02208-1.

## Introduction

Chronic obstructive pulmonary disease (COPD) is an inflammatory disease characterized by irreversible airflow limitation due to obstructive bronchiolitis, emphysema, and chronic pulmonary inflammation [1]. It’s the third leading cause of death worldwide [2] and it’s estimated that 10% of adults over 40 currently live with COPD [3]. COPD results from long-term exposure to harmful gases and particles, combined with individual factors including genetic susceptibility, nutrition and aging. The main etiologic factor is smoking, but also indoor air pollution, and occupational dusts, fumes and chemicals were identified as important risk factors [2].

Although rarely found in healthy lungs, the presence of B cells in lungs is associated with infection and chronic inflammatory conditions such as COPD [4]. Autoantibodies have been found in COPD patients [5] and severe stages of the disease are characterized by increased B cell numbers and B cell-rich lymphoid follicles around the small airways [6, 7]. In addition, levels of the B cell attracting chemokine CXCL13 and the B cell survival/maturation factor BAFF have been shown to be increased in lungs from COPD patients [8, 9]. Thus, dysfunctional B cells and their products are considered to play a pathogenic role in COPD.

Beyond their role as antibody producing cells, other important roles such as antigen presentation and cytokine production have been attributed to B cells. Based on their cytokine production profile, two opposing sets of B cells have been defined: effector B cells (B-effs) positively regulating immune responses through release of pro-inflammatory cytokines such as interleukin (IL)-6, IFN-γ, and GM-CSF versus regulatory B cells (B-regs) that negatively regulate immune responses through release of anti-inflammatory cytokines such as IL-10, IL-35, and transforming growth factor (TGF)-β [10]. B-regs function as a feedback mechanism that maintains the immune balance by preventing excessive inflammation and tissue damage. Decreased and/or impaired B-regs have been observed in many autoimmune diseases, infectious diseases, and cancers leading to an immune imbalance [10–13].

B cell derived IL-10 is the hallmark for the identification of B-regs in mice and human [14, 15]. B-reg derived IL-10 is responsible for conversion of CD4 + T cells into regulatory T cells (Tregs), inhibition of Th1/Th17 differentiation, inhibition of TNF-α production by monocytes, and maintenance of invariant natural killer cells (iNKT) [16]. In this study, we defined B cell subsets with regulatory capabilities through IL-10 production as IL10 + B-regs. The approach of BAFF inhibition to restore B-reg/B-eff imbalance is unexplored for COPD and might be worthwhile exploring since local expansion of B-regs in the lung has been proposed [16]. Research on transcription factors involved in IL-10 production is inconclusive [12], but Hypoxia Inducible Factor 1 subunit alpha (HIF-1α) [17] and Interferon Regulatory Factor 4 (IRF4) [18] have been identified as promotors of IL-10 production.

The aim of this research was to study regulatory and effector B cells in COPD to provide a scientific basis for potential therapies targeting B cells in COPD. First, we verified the presence of IL-10 producing B-regs in human lung tissue. Secondly, we quantified IL-10 producing B-regs and IL-6 producing B-effs in peripheral blood mononuclear cells (PBMCs) from healthy controls, smokers without airflow limitation, and COPD patients. Thirdly, we exposed B cells from COPD patients in vitro to CSE and quantified IL-10 producing B-regs and IL-6 producing B-effs. Next, we blocked BAFF in these in vitro CSE exposed B cells aiming to restore IL-10 production. Fourthly, we quantified mRNA levels of transcription factors IRF4 and HIF-1α in naive and memory B cells exposed to medium or CSE.

## Research design and methods

Data are reported according to the STROBE checklist for cross-sectional studies [19, 20].

### Immunohistochemical staining for IL-10 and CD20 in human lung tissue

#### Human lung tissue samples for IHC

Immunohistochemical (IHC) staining was performed on lung resection specimens obtained from 5 patients at Ghent University Hospital (Belgium). Patient characteristics from the subject enrolled can be found in Additional file 1: Table S1. Lung tissue was derived from patients diagnosed with solitary pulmonary tumors at maximum distance from the pulmonary lesions and without signs of retro-obstructive pneumonia or tumor invasion. None of the surgical patients were treated with neo-adjuvant chemotherapy. All samples were collected between 2002 and 2020 in a non-probabilistic manner. Patients provided written informed consent. This study was approved by the medical ethical committee of Ghent University Hospital (2011/0114; 2016/0132; 2019/0537).

#### IHC staining procedure

Paraffin-embedded lung tissue sections first underwent antigen retrieval with citrate buffer (Scytek), followed by incubation with anti-IL10 antibody for 24 h. Next, slides were colored with AF488 conjugated donkey-anti-rat secondary antibody for 2 h. Slides were then incubated with mouse anti-human CD20 antibody for 2 h, followed by coloring with AF647 conjugated donkey-anti-mouse secondary antibody and DAPI. Appropriate isotype controls were used during the staining procedure to test for non-specific interactions. The antibodies used for immunohistochemistry can be found in Additional file 1: Table S2.

### Quantification of B cell subsets and IL-6- and IL-10 producing B cells in blood

#### Study population

Characteristics of subjects (n = 30) that were included in the PBMC isolation are presented in Table 1. The samples from 5 COPD patients used for B cell purification following PBMC isolation used during the in vitro CSE-exposure experiment are part from the same cohort.

**Table 1:** Characteristics of subjects enrolled in the quantification of IL-10 producing B-regs in PBMCs (n = 30)

	Healthy	Smoker	COPD
Subjects	N = 9	N = 8	N = 13
Age (years)	57.22 (6.18)	62.63 (7.44)	63.54 (6.79)
Male sex (%)	2 (22.22)	3 (37.5)	6 (46.2)
BMI	26.11 (5.78)	27.02 (3.31)	25.82 (5.82)
Smoking			
Ex (%)	NA	4 (50)	10 (76.9)
Current (%)	NA	4 (50)	3 (23.1)
Pack-years	0 (0–0)	24,5^+^ (12.80–36,20)	36.12^+^ (28.42–43.80)
Lung function			
FEV1 pre (% predicted)	108 (97–118)	106 (95–118)	46^+ϒ^ (32–61)
FEV1 post (% predicted)	NA	NA	44 (31–56)
FVC pre (% predicted)	112 (102–123)	109 (96–121)	82 (71–94)
FEV1/FVC pre (%)	77 (74–79)	78 (75–81)	43^+ϒ^ (33–53)
FEV1/FVC post (%)	NA	NA	41 (31–51)
CAT test score total	8.22 (2.81)	8.75 (5.75)	19.08^ο⊥^ (8.62)
Medication			
SABA/SAMA (yes/no)	0/9	0/8	7/6
LABA/LAMA (yes/no)	1/8	0/8	12/1
ICS (yes/no)	1/8	0/8	8/5
Azithromycin (yes/no)	0/9	0/8	3/10
OCS (yes/no)	0/9	0/8	0/13
White blood cell differentiation			
Hemoglobin (g/dL)	13.94 (0.93)	13.73 (1.52)	14.32 (1.12)
White blood cells (10E3/μL)	6.45 (1.55)	7.34 (1.71)	7.64 (1.54)
Neutrophil (%)	58.82 (6.83)	62.84 (4.59)	64.72 (11.55)
Lymphocytes (%)	32.87 (6.74)	27.99 (4.10)	26.49 (10.77)
Monocytes (%)	7.46 (2.51)	7.34 (2.03)	6.67 (1.94)
Eosinophils (%)	2.24 (1.02)	1.36 (0.72)	1.73 (1.12)
Basophils (%)	0.51 (0.24)	0.42 (0.21)	0.38 (0.21)

COPD (Chronic Obstructive Pulmonary Disease); BMI (body mass index); NA (not applicable); FEV1 pre/post (forced expiratory volume 1 s pre/post-bronchodilator); FVC pre (forced vital capacity pre-bronchodilator); SABA/SAMA (short-acting beta agonist/short-acting muscarin antagonist); LABA/LAMA (long-acting beta agonist/long-acting muscarin antagonist); ICS (inhalation corticosteroids); OCS (oral corticosteroids). Data are represented as mean (standard deviation or 95% confidence interval). ^+^P < 0.05 versus healthy ^ϒ^P < 0.05 versus smoker ^ο^P < 0.01 versus healthy ^⊥^P < 0.01 versus smoker

#### Human patient cohort for PBMC and B cell isolation

Blood from 30 subjects recruited at Ghent University Hospital (Belgium) was used for quantification of IL-10 + B-regs and IL-6 + B-effs. Subjects were categorized as healthy controls, smokers without airflow limitation, or COPD patients based on medical- and smoking history, questionnaires and spirometry. Subjects were considered ex-smokers when they had quit smoking for at least 1 year. COPD severity was defined according to the Global Initiative for Chronic Obstructive Lung Disease (GOLD) classification. Patients provided written informed consent and the study was approved by the medical ethical committee of Ghent University Hospital (2016/0463).

PBMCs were isolated from whole blood using a Ficoll Paque Plus (GE Healthcare) density gradient in Leucosep tubes (Greiner Bio). PBMCs were used as such for quantification of IL10 + B-regs and IL-6 + B-effs in different subject groups (never smoker, smokers, and COPD patients). Blood-derived B cells were used for quantification of IL-10 + B-regs and IL-6 + B-effs upon in vitro exposure to cigarette smoke extract (CSE). These were obtained by positive selection of CD19 + cells by magnetic separation (Easysep^®^ Human B cell enrichment kit, STEMCELL Technologies) of PBMCs from 5 COPD patients.

#### Extracellular cytokine staining

B cell subsets were identified by flow cytometry on PBMCs based on their extracellular expression profile. To minimize non-specific binding, single-cell suspensions were first incubated with FcR blocking antibody, followed by labeling reactions to identify B cell subsets. The antibodies used for identification of B cells can be found in Additional file 1: Table S2. FlowJo Software (Tree Star Inc, Ashland, USA) was used to analyze the data. An example of the gating strategy used can be found in Additional file 2: Figure S1.

#### PBMC and B cell stimulation

PBMCs or B cells (2 × 10^5^ cells) were seeded in a 96-well plate in RPMI 1640 medium (Gibco) supplemented with 10% Fetal Bovine Serum (Thermo Fisher Scientific), 100 U/mL penicillin and 100 µg/mL streptomycin (both from Merck). Cells were stimulated for 48 h with recombinant human CD40L (1 µg/mL, R&D systems) and TLR9 agonist (0.77 µg/mL, ODN2006-TLR9 agonist CpG Class B, Invivogen) with addition of eBioscience™ Cell Stimulation Cocktail (plus protein transport inhibitors, Thermo Fisher Scientific) during the final 4 h of stimulation.

During the 48 h stimulation period, magnetically sorted B cells were cultured in 5% and 10% cigarette smoke extract (CSE). Cigarette smoke extract was obtained by passing the smoke of 10 cigarettes through 30 mL cell culture medium. After sterile filtering (Nalgene rapid-flow filter unit—Thermo Fisher Scientific) of the stock solution, a 1/20 or 1/10 dilution resulted in a final concentration of 5% or 10% CSE respectively. To neutralize BAFF upon in vitro CSE exposure, recombinant human BAFFR-Fc chimera protein (500 ng/mL, R&D systems) was added during the 48 h stimulation period.

#### Intracellular cytokine staining

Flow cytometric detection of intracellular cytokine production was performed on stimulated PBMCs and B cells. To minimize non-specific binding, single-cell suspensions were first incubated with FcR blocking antibody, followed by labeling reactions to identify IL-6- and IL-10- producing B cells within total B cells and within the different B cell subsets: naive B cells (CD27 − IgD + IgM +), natural effector cells (CD27 + IgD + IgM +), activated memory B cells (CD27 + IgD-IgM +), and class-switched memory B cells (CD27 + IgD − IgM −). The antibodies used for can be found in Additional file 1: Table S2. FlowJo Software (Tree Star Inc, Ashland, USA) was used to analyze the data. An example of the gating strategy to detect IL-10 + and IL-6 + B cells can be found in Additional file 3: Figure S2.

### Gene expression of IL-10 transcription factors IRF4 and HIF-1α in B cells

#### Study population

Characteristics of subjects (n = 19) that were included in the PBMC isolation followed by B cell purification and sorting into naive- and memory B cells are presented in Additional file 1: Table S3.

#### Human patient cohort for B cell isolation

Peripheral blood mononuclear cells (PBMCs) were isolated from whole blood from 19 subjects recruited at Ghent University Hospital (Belgium) and used for flowcytometric sorting into naive and memory B cells. Patients provided written informed consent and the study was approved by the medical ethical committee of Ghent University Hospital (2016/0463).

#### Exposure of naive- and memory B cells to medium or cigarette smoke extract

Enrichment of B cells was performed on PBMC using the Easysep^®^ Human B cell enrichment kit (stem cell technologies) followed by flow cytometrically sorting into naive B cells (defined as CD27 − IgD + cells) and memory B cells (defined as CD27 + IgD − cells) and exposure to medium or CSE for 48 h. After 48 h, RNA was extracted miRNeasy micro kit (QIAGEN), according to the manufacturer’s instructions. Details on bulk RNA sequencing and processing can be found in the Additional file 1.

### Statistical analysis

Statistical analysis was performed with IBM SPSS Statistics (version 27), using Student’s T-test for comparing parametrical or a one-way ANOVA with Bonferroni correction for continuous variables, Mann–Whitney U test or Kruskal Wallis test for comparing non-parametrical continuous variables, and Fisher’s exact test for comparing categorical variables. Normality was confirmed using a Shapiro–Wilk Normality test. A Pearson correlation coefficient was calculated for linear associations in scatter plots. p-values < 0.05 were considered statistically significant.

## Results

### Immunohistochemical staining for IL-10 and CD20 in human lung tissue

**Fig. 1:**
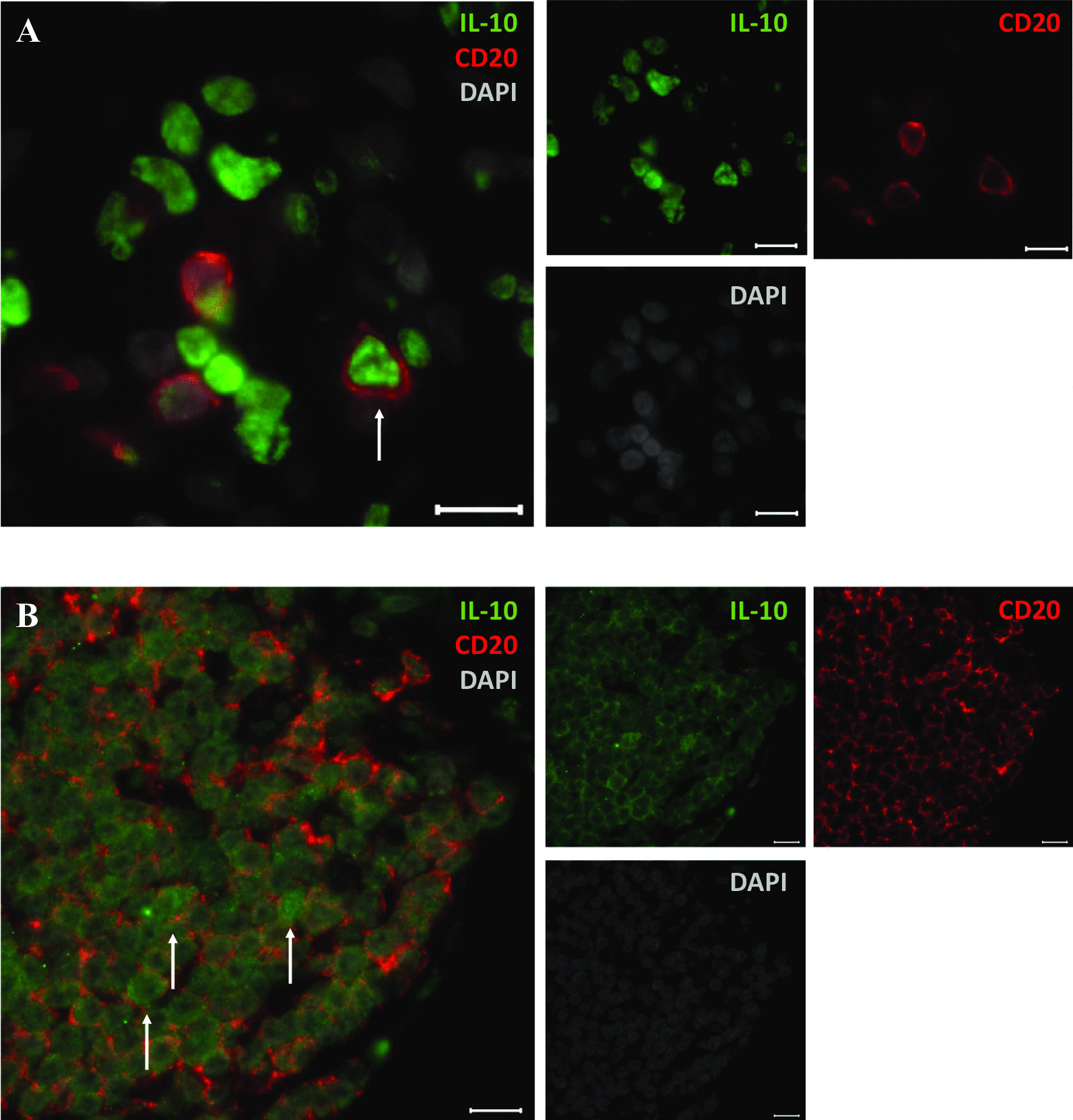
Immunohistochemistry (IHC) of IL-10 producing regulatory B cells in human lung tissue. Representative images of IL-10 + CD20 + double positive immunofluorescent staining showing positive signal in **A** human lung parenchyma within proximity of an airway, and **B** lymphoid follicles found in human lung samples. All images were taken at a 630 × magnification. Small images are individual channel results. White arrows point towards IL-10 + CD20 + double positive cells

The presence of IL-10 producing regulatory B cells in lung tissue (n = 5) was confirmed by immunohistochemistry. Double positive cells for IL-10 (green) and CD20 (red) were observed in lung parenchyma within proximity of an airway (Fig. 1a), and within lymphoid follicles (Fig. 1b). No signal for both CD20 and IL-10 could be observed in isotype control stained tissue (Additional file 4: Figure S3).

### Quantification of B cell subsets and IL-6- and IL-10 producing B cells in blood

#### Identification of B cell subsets in human PBMCs

**Fig. 2:**
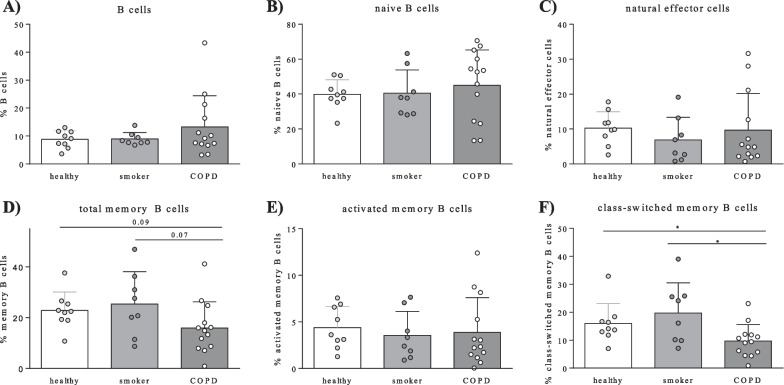
B cell subtypes in peripheral blood from healthy controls, smokers, and COPD patients determined by flow cytometry. Percentages of different B cell subsets within different groups (healthy controls, smokers without airway obstruction, and COPD patients) are depicted: **A** CD19 + CD20 + alive B cells within alive lymphocytes, **B** naive B cells (CD27-IgD + IgM −) within total B cells, **C** natural effector cells (CD27 + IgD + IgM +) within total B lymphocytes, **D** total memory B cells (CD27 + IgD −), **E** activated memory B cells (CD27 + IgD − IgM +), and **F** class-switched memory B cells (CD27 + IgD – IgM −) within total B lymphocytes

Total B cell percentages and proportions of the different B cell subsets [naive B cells (CD27 − IgD + IgM +), natural effector cells (CD27 + IgD + IgM +), total memory B cells (CD27 + IgD −), activated memory B cells (CD27 + IgD − IgM +), and class-switched memory B cells (CD27 + IgD − IgM −)] in healthy controls, smokers without airway obstruction, and COPD patients (GOLD stages I–IV) are shown in Fig. 2. Furthermore, no significant differences between early-stage B cell subsets (naive B cells, natural effector cells) were observed, whereas memory B cells were significantly decreased in COPD patients compared to non-COPD controls (data not shown, p-value: 0.022). Furthermore, class-switched memory B cells were significantly decreased in COPD patients compared to both healthy controls (p-value: 0.017) and smokers without airway obstruction (p-value: 0.030, Fig. 2f).

#### Identification of IL-10 producing B-regs and IL-6 producing B-effs in human PBMCs

**Fig. 3:**
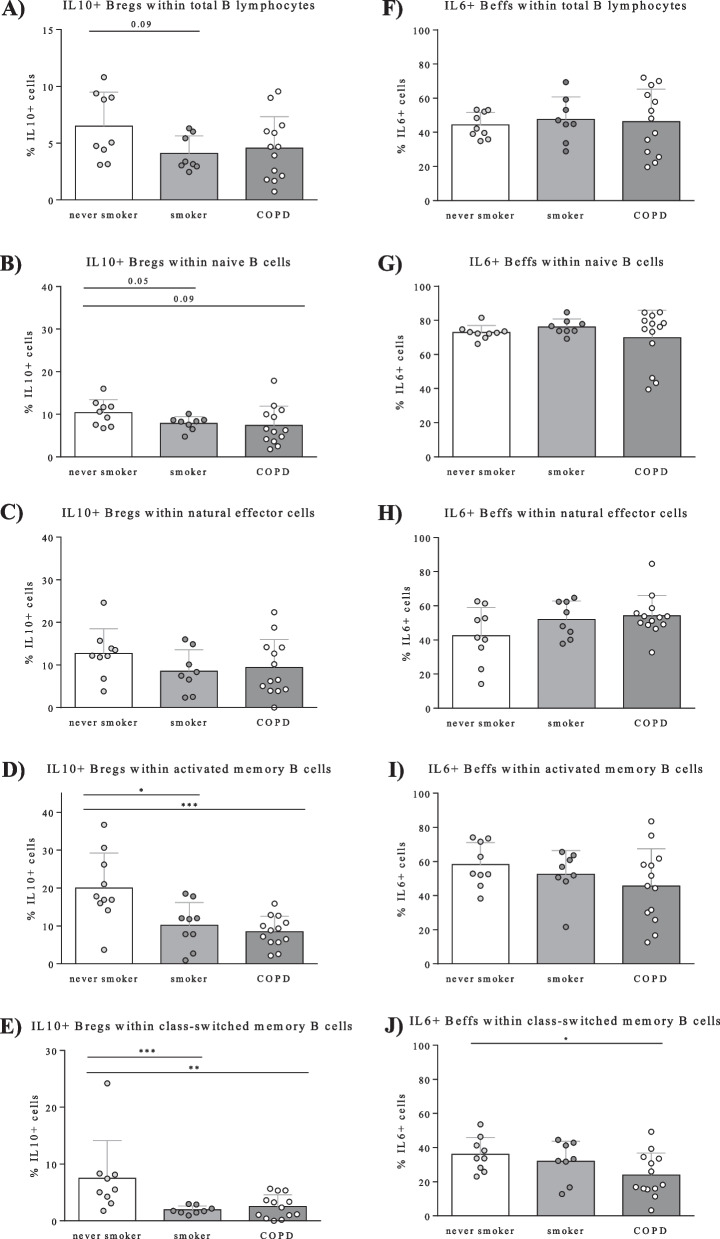
Percentages of IL-10 producing B-reg cells and IL6 producing B-eff cells in peripheral blood from healthy controls, smokers, and COPD patients determined by flow cytometry. Percentages of IL-10 producing cells (**A**–**E**) and IL-6 producing cells (**F**–**J**) within total B lymphocytes and B cell subsets within different groups (healthy controls, smokers without airway obstruction, and COPD patients) are depicted

Percentages of IL-10 + B-regs within total B lymphocytes and within the different B cell subsets determined by flow cytometry are shown in Fig. 3a–e. Within total B cells and B cell subsets, percentages of cells producing IL-10 were attenuated in smokers without airway obstruction and patients with COPD, compared to healthy controls. This attenuation reached statistical significance in total memory B cell (data not shown) and both subtypes (activated memory B cells and class-switched memory B cells) as shown in Fig. 3d, e.

Percentages of IL-6 + B-effs within total B lymphocytes and within the different subsets are depicted in Fig. 3f–j. No differences were observed in IL-6 + B-effs within total B lymphocytes and early-stage B cell subsets (naive B cells, natural effector cells) from smokers without airway obstruction and COPD patients, compared to healthy controls. In memory B cell subtypes, the percentages of IL-6 + B-effs in class-switched memory B cells were significantly decreased in COPD patients, compared to healthy controls (Fig. 3j).

The ratio of IL-6 producing cells to IL-10 producing cells within total B lymphocytes and within all the B cell subsets (Additional file 5: Figure S4a-e) was increased in smokers and COPD patients, compared to healthy controls and reached statistical significance in smokers within the total B lymphocyte, natural effector-, and class-switched memory B cell populations. Additionally, COPD patients had significantly increased IL6/IL10 ratio’s compared to healthy controls in natural effector cells (Additional file 5: Figure S4c).

**Fig. 4:**
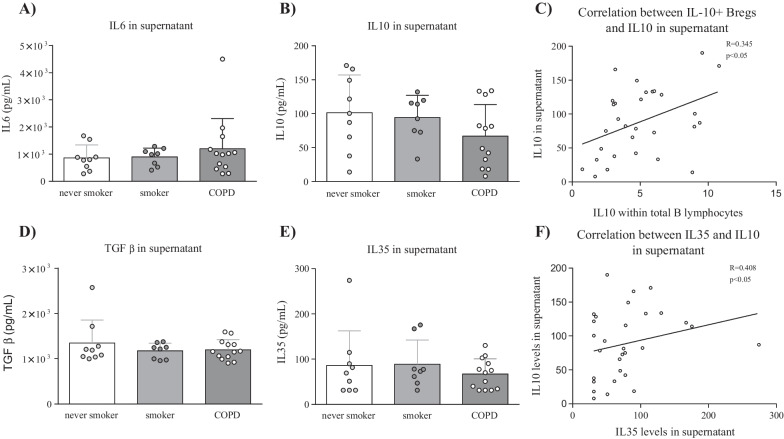
IL-6, IL-10, IL-35, and TGF-β levels in cell culture supernatant of stimulated PBMCs. Levels of different pro- and anti-inflammatory cytokines were determined in cell culture supernatant that was obtained after the 48 h in vitro stimulation period. Levels of different cytokines in healthy controls, smokers without airway obstruction, and COPD patients were determined: **A** levels of IL-6 in cell-culture supernatant, **B** levels of IL-10 in cell-culture supernatant, **C** Correlation between IL-10 levels in supernatant and IL-10 + B-regs in total B lymphocytes, **D** levels of TGF-β in cell-culture supernatant, **E** levels of IL-35 in cell-culture supernatant, and **F** Correlation between IL-10 and IL-35 levels in cell-culture supernatant

Levels of the pro-inflammatory cytokine IL-6 and the anti-inflammatory cytokines IL-10, IL-35, and TGF-β were determined in supernatant of stimulated PBMCs by ELISA (Fig. 4). IL-6 levels were numerically increased in cell-culture supernatant from COPD patients, compared to smokers and healthy controls (Fig. 4a). IL-10 and IL-35 supernatant levels were not significantly different in COPD patients compared to healthy controls (Fig. 4b, e). However the IL-10 and IL-35 levels correlated significantly with each other and with IL-10 + B-regs present within the total B lymphocyte population (Fig. 4c, f). Levels of TGF-β did not differ between different subject groups (Fig. 4d).

#### The effect of in vitro exposure to cigarette smoke extract on the capacity of B cells to produce IL-10 and IL-6

**Fig. 5:**
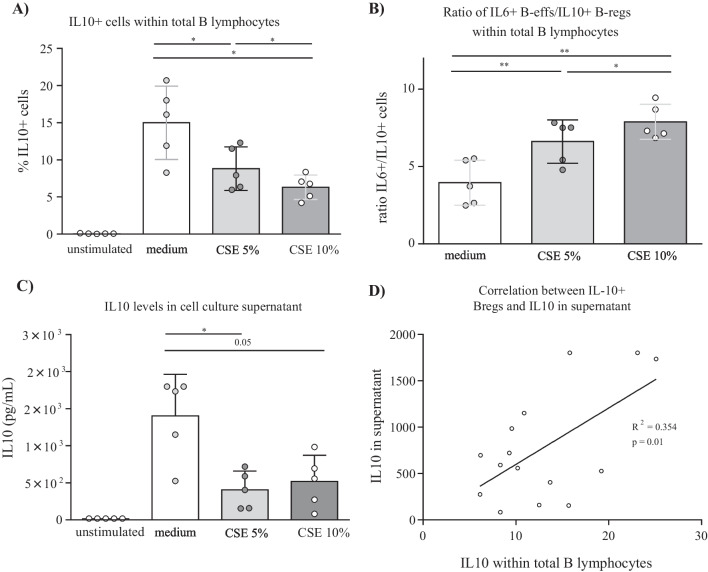
Effect of in vitro CSE-exposure on the capacity of B cells to produce IL-10. The capacity of MACS sorted B cells to produce IL-10 upon CSE-exposure was determined by flow cytometry: **A** Percentages of IL-10 producing cells within total B lymphocytes in non-stimulated cells and stimulated cells present in medium, 5% cigarette smoke extract (CSE), and 10% CSE, **B** IL6 + /IL10 + percentages in stimulated cells present in medium, 5% CSE, and 10% CSE. The levels of IL-10 in supernatant obtained after the stimulation period were measured by ELISA: **C** Levels of IL-10 in cell culture supernatant, and **D** Correlation between IL10 levels in supernatant determined by ELISA and IL10 + cells determined by flow cytometry

First, the capacity of magnetically sorted and stimulated B cells to produce IL6 and IL10 upon exposure to 5% and 10% CSE was determined by flow cytometry. B cells that were cultured in medium without stimulation with CD40L and the TLR9 agonist CpG for 48-h, and without the addition of phorbol 12-myristate 13-acetate (PMA), ionomycin, and brefeldin (PIB) during the final 4 h, did not produce IL-10. The capacity of stimulated B cells to produce IL-10 was significantly reduced upon exposure to CSE compared to its medium exposed counterparts, and this in a concentration-dependent manner (Fig. 5a). The capacity of B cells to produce IL6 was not significantly altered when cells were exposed to CSE (data not shown). Additionally, the IL6 + cells/IL10 + cells ratio was significantly increased upon exposure to CSE (Fig. 5b). Secondly, IL-10 levels measured by ELISA were significantly reduced in the supernatant from CSE exposed B cells (Fig. 5c) and correlated significantly with the percentage of IL-10 + cells (Fig. 5d), which confirmed our results found via flow cytometry.

In a follow-up experiment, 500 ng/mL BAFF-Fc-Chimera was added during stimulation and this did not change the percentage of IL10 + B cells (Additional file 6: Figure S5a) or IL6 + B cells (data not shown) within total B lymphocytes. The IL10 levels in supernatant determined by ELISA (Additional file 6: Figure S5b) are in line with the flow cytometry data and did not differ upon BAFF inhibition.

### Gene expression of IL-10 transcription factors IRF4 and HIF-1α in B cells

**Fig. 6:**
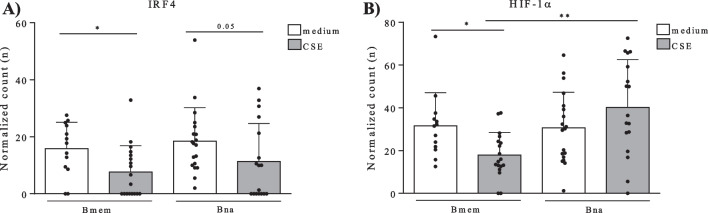
mRNA levels of transcription factors IRF4 and HIF-1α in sorted naive- and memory B cells upon medium or CSE exposure. mRNA levels of IL-10 transcription factors in FACS sorted naive- and memory B cells exposed to medium- or CSE were determined by bulk RNA sequencing: **A** IRF4 normalized counts in memory B cells (Bmem) and naive B cells (Bna) upon medium (white bars) or 5% cigarette smoke extract (CSE, grey bars) exposure, and **B** HIF-1α normalized counts in memory B cells (Bmem) and naive B cells (Bna) upon medium (white bars) or 5% CSE (grey bars) exposure

Normalized counts of IL-10 transcription factors IRF4 and HIF-1α in flow cytometric sorted naive- (IgD + CD27 −) and memory (CD27 + IgD −) B cells upon medium- and CSE exposure are depicted in Fig. 6. In memory B cells (Bmem), mRNA levels of both transcription factors were significantly decreased upon CSE exposure, compared to medium exposed cells. In naive B cells (Bna), IRF4 levels where significantly decreased upon CSE exposure whereas HIF-1α levels tended to increase upon CSE exposure, but the latter did not reach statistical significance. Furthermore, HIF-1α levels were significantly higher in CSE exposed Bna compared to CSE exposed Bmem.

## Discussion

In this study, for the first time, we have demonstrated the presence of IL10 producing B-regs in human lung tissue from COPD patients. Additionally, decreased percentages of IL-10 + B-reg cells in total memory B cells and its subsets were observed in peripheral blood of smokers without airflow limitation and patients with COPD, compared to never smokers. Next, addition of cigarette smoke extract (CSE) to B cells resulted in reduced capacity to produce IL-10. Furthermore, decreased levels of transcription factors involved in IL-10 production (IRF4 and HIF-1α) upon CSE exposure may provide a mechanistic explanation for the observed results.

No differences in percentages of B cells and its early stage subsets (naive B cells, natural effector cells) in peripheral blood were observed between patient groups, whereas class-switched memory B cell proportions were significantly lower in COPD patients compared to healthy controls and smokers without airway obstruction. These results differ from previously published results where a decrease of class-switched memory B cells was observed in current smokers, irrespective of COPD status [21]. This discrepancy might be explained by the limited number of current smokers within the COPD patients in our study (n = 3 within a total of 13 COPD patients) or the use of different definitions for ‘current smokers’. However, total memory B cells (defined in the article [21] as CD20 + CD27 +) were significantly reduced in COPD patients compared to non-COPD controls, a result that is confirmed by our data. Resident memory B cells are a common feature of antigen-exposed lungs [22, 23] and it is therefore tempting to speculate that massive influx of memory B cells into the lung compartment results in their depletion in blood.

B cells at any stage of development can be activated to secrete IL-10 [16] by both the inflammatory environment and intracellular interaction [24]. Decreased percentages of IL-10 + B-regs were observed in peripheral blood of smokers without airway obstruction and COPD patients, compared to healthy controls. This decrease reached statistical significance in total memory B cells and within both memory B cell subsets (activated-, and class-switched memory B cells). Furthermore, B-eff/B-reg ratio’s increased in smokers and COPD patients compared to healthy controls suggestive of an imbalance between these two B cell subtypes. Previous research has shown decreased IL-10 levels and increased IL-6/IL-10 ratio’s in bronchial secretions and serum of COPD [25, 26] but since many inflammatory cells (monocytes, macrophages, mast cells, and T-& B-lymphocytes) can produce IL-10 [27], we can now attribute the observed decrease in IL-10 to decreased production by B-reg cells. Furthermore, a B-reg/B-eff imbalance has been observed in many inflammatory and auto-immune diseases [11, 28–31] and our results show that this is also true for COPD. A recent publication by *Lin and colleagues* [32] shows decreased levels of regulatory B cells in COPD patients, a finding that is confirmed in the present study. However, we used IL-10 production upon stimulation to define regulatory B cells whereas *Lin and colleagues* mainly use surface marker expression, which is not the optimal strategy to define B-regs since this only identifies one subset of B-regs [33].

In a follow-up experiment, we determined the effect of CSE-exposure on the capacity of B cells and its subsets to produce IL-6 and IL-10 to verify whether cigarette smoke has a direct effect on B cell derived IL-10 production. In this experiment, a concentration-dependent reduction in B cell capacity to produce IL-10 was observed suggesting the presence of a direct effect. Furthermore, mRNA levels of transcription factors involved in IL-10 production (IRF4 and HIF-1α) decrease in memory B cells upon CSE exposure, which could explain the decreased levels found in this cell type in smokers and COPD patients since these patients are chronically exposed to cigarette smoke. Additionally, the observed decrease in transcription factors upon CSE exposure, could also explain the reduced capacity of B cells to produce IL-10 upon CSE exposure that was observed in the second experiment. Altogether, these finding might increase insights into COPD pathogenesis advocating a role for the B-eff/B-reg imbalance early in disease onset since reduced IL-10 production is not only observed in COPD patients but also in smokers and upon smoke-exposure. IL-10 expression can be induced by signals such as toll-like-receptors (TLR) ligands [34], CD40-ligand (CD40-L), inflammatory cytokines (IL-6, IL-1β, BAFF), and co-stimulatory molecules. We have not been able to show restored capacity of B cells to produce IL-10 by addition of 500 ng/mL BAFF-Fc-Chimera opposed to previous research performed in scleroderma [30], where BAFF inhibition restored B-eff/B-reg imbalances in mice.

In this study, we show decreased numbers and function of IL-10 producing B-regs in peripheral blood from both smokers and COPD patients. The main strengths of the study are the use of IL-10 as a marker for B-reg cells combined with a 48-h stimulation protocol allowing quantification of total B-regs [24, 35] and the concomitant determination of IL-6 production providing insights into B-reg/B-eff balances. Furthermore, the addition of various cell surface markers allowed in depth characterization of the capacity of different B cell subsets to produce IL-10 upon stimulation. However, the sample size of our patient cohort is relatively small and confirmation of our results in larger patient cohorts, preferably consisting of more current smokers within the COPD patient group, is needed. The main limitation of this study is that the major part of it was performed on blood-derived samples and additional studies in lung tissue, a more relevant compartment for COPD research, are necessary. More specifically, quantification of IL10 + B-regs in lung tissue of patients with COPD is needed to confirm the blood-derived data. Furthermore, a part of the cytokine profiling is performed on supernatant from PBMC cultures and therefore we cannot attribute these measurements specifically to B cells as other cell types present might contribute to the cytokine levels measured. Research on transcription factors responsible for IL-10 production is currently limited [12, 17, 18] and additional studies to confirm the role of IRF4 and HIF-1α in IL-10 production and identification of other transcription factors that are involved in IL-10 production is needed to further support our results.

In conclusion, we show decreased numbers and function of IL-10 producing regulatory B cells upon smoke-exposure and in COPD patients and smokers. Since smoking is the main risk factor for COPD, this impaired B-reg function in smokers and patients with COPD might contribute to the initiation and progression of the disease.

## Supplementary Information

**Additional file 1. **Supplementary Information. Written supplementary information provided in addition to the main text of the research paper.

**Additional file 2. Supplementary Figure 1. **Gating strategy to identify B cell subsets. Representative example of the gating strategy used to define the following subsets: A) lymphocytes within single cell populations, B) alive CD20+ B cells within lymphocytes, C) CD27 and IgD expression within the total B cells population, D) naïve B cells (CD27-IgD+IgM+), E) natural effector cells (CD27+IgD+IgM+), and F) activated memory B cells (CD27+IgD-IgM+) and class-switched memory B cells (CD27+IgD-IgM-).

**Additional file 3. Supplementary Figure 2. **Gating strategy to determine IL-10 and IL-6 expression. Representative example of the gating strategy used to determine IL-6+ and IL-10+ cells within total B cells and the different B cell subsets as defined in Supplementary Figure 2.

**Additional file 4. Supplementary Figure 3. **IHC stain for IL-10 and CD20 with isotype control. Representative image of IL-10 and CD20 immunohistochemical staining showing A) positive signal for IL-10 (green), CD20 (red), and nuclei (grey), B) negative signal when the tissue section was stained with isotype controls. All images were taken at a 630x magnification. Scale bar length is 10 μm.

**Additional file 5. Supplementary Figure 4. **Ratios of IL6+ B-effs to IL10+ B-regs in peripheral blood from healthy controls, smokers, and COPD patients determined by flow cytometry. Ratios of IL6+ B-effs to IL10+ B-regs within total B lymphocytes (A) and B cell subsets (B-E) are shown.

**Additional file 6. Supplementary Figure 5. **Capacity of magnetically sorted B cells to produce IL-10 upon cigarette-exposure with BAFF inhibition. The capacity of MACS sorted B cells to produce IL-10 upon cigarette smoke exposure with and without the addition of BAFF-Fc-Chimera (inhibition of BAFF) was determined by flow cytometry and ELISA: A) Percentages of IL10+ cells within unstimulated cells, stimulated cells in medium, 5% CSE, and 10% CSE without the addition of BAFF-Fc-Chimera (white) and with the addition of BAFF-Fc-Chimera (grey) determined by flow cytometry, B) Levels of IL-10 in supernatant of stimulated cells in medium, 5% CSE, and 10% CSE without the addition of BAFF-Fc-Chimera (white) and with the addition of BAFF-Fc-Chimera (grey) determined by ELISA.

## Data Availability

The datasets used and/or analysed during the current study are available from the corresponding author on reasonable request.

## Abbreviations

BAFF: B cell-activating factor
BAFFR: B cell-activating factor receptor
B-effs: Effector B cells
Bmem: Memory B cells
Bna: Naive B cells
B-regs: Regulatory B cells
CD: Cluster of differentiation
COPD: Chronic Obstructive Pulmonary Disease
CSE: Cigarette smoke extract
GM-CSF: Granulocyte–macrophage colony-stimulating factor
GOLD: Global Initiative for Chronic Obstructive Lung Disease
HIF-1α: Hypoxia Inducible Factor 1 subunit alpha
IFN-γ: Interferon gamma
IHC: Immunohistochemical staining
iNKT: Invariant natural killer cell
IL-6: Interleukin 6
IL-10: Interleukin 10
IL-35: Interleukin 35
IRF4: Interferon Regulatory Factor 4
PBMC: Peripheral blood mononuclear cells
TGF-β: Transforming growth factor beta
Th1: T helper 1 cell
Th17: T helper 17 cell
TLR9: Toll like receptor 9
