# Supplementary material for: IL-10 producing regulatory B cells are decreased in blood from smokers and COPD patients
Source: Respir Res. 2022 Oct 17;23:287. doi: 10.1186/s12931-022-02208-1 (PMC9578234; doi:10.1186/s12931-022-02208-1)
Supplement: Supplementary file 1 — Additional file 1. Supplementary Information. Written supplementary information provided in addition to the main text of the research paper. [file 12931_2022_2208_MOESM1_ESM.docx]

**Supplementary Information**

**IL-10 producing regulatory B cells are decreased in blood from smokers and COPD patients**

Merel Jacobs^1^, Sven Verschraegen^2^, Bihiyga Salhi^2^, Jasper Anckaert^3^, Pieter Mestdagh^3^, Guy G Brusselle^1,2,4,5^, Ken R Bracke^1^

^1^Laboratory for Translational Research in Obstructive Pulmonary Diseases, Department of Respiratory Medicine, Ghent University Hospital, Ghent, Belgium.

^2^Department of Respiratory Medicine, Ghent University Hospital, Ghent, Belgium.

^3^Center for Medical Genetics, Department of Biomolecular Medicine, Ghent University, Ghent, Belgium; OncoRNALab, Cancer Research Institute Ghent (CRIG), Ghent University, Ghent, Belgium.

^4^Department of Epidemiology, Erasmus Medical Center, Rotterdam, The Netherlands

^5^Department of Respiratory Diseases, Erasmus Medical Center, Rotterdam, The Netherlands

**Corresponding author:** Prof. Ken Bracke, Department of Respiratory Medicine, Ghent University Hospital, C. Heymanslaan 10, 9000 Ghent, Belgium, ken.bracke@UGent.be

**Keywords:** regulatory B cells, COPD (chronic obstructive pulmonary disease), cigarette smoke, IL-10 (interleukin-10)

**RESEARCH DESIGN AND METHODS**

**Gene expression of IL-10 transcription factors IRF4 and HIF-1α in B cells**

*Bulk RNA sequencing and processing*

PolyA+ RNA sequencing libraries were prepared using the QuantSeq 3‘ mRNA-Seq Library Prep Kit (Lexogen) according to the manufacturer’s instructions. Using 40 ng of RNA input, libraries were quantified and pooled for single-end sequencing on a NextSeq 500. RNA sequencing quality control was performed using FASTQC (0.11.8) and reads were mapped to the human reference genome (GRCh38 build) using STAR (2.6.0c), followed by calculation of the counts using HTSeq (0.11.0). Genomic features with a zero read count across all samples and with mean read count below 10 across samples were discarded. Normalization of the counts was performed using the R statistical programming language (64 Bit, version 4.1.0) and the DESeq-2 package (1.32.0). Normalized counts for IRF4 and HIF-1α were plotted across different conditions. More in depth analyses of this dataset will be performed and published elsewhere.

**TABLES**

**Supplementary Table 1. Characteristics of subjects enrolled in the determination of IL-10 producing Bregs in lung tissue (n=5).**

|  |  | **Subject 1** | **Subject 2** | **Subject 3** | **Subject 4** | **Subject 5** |
| --- | --- | --- | --- | --- | --- | --- |
|  | **Age (years)** | 52 | 73 | 58 | 58 | 64 |
|  | **Sex** | female | male | male | male | female |
|  | **BMI** | 23 | 28 | 25 | 28 | 23 |
|  | **Smoking** |  |  |  |  |  |
|  | Smoking status (CS/ES/NS) | CS | ES | NS | CS | CS |
|  | Packyears | 40 | 55 | 0 | 25 | 38 |
|  | **Lung function** |  |  |  |  |  |
|  | COPD status | no | GOLD II | no | no | COPD II |
|  | FEV1 pre (% predicted) | 83 | 54 | 114 | 90 | 60 |
|  | FEV1 post (% predicted) | 83 | 56 | 114 | 90 | 64 |
|  | FVC post (% predicted) | 99 | 96 | 99 | 80 | 114 |
|  | FEV1/FVC pre (%) | 66 | 47 | 88 | 87 | 44 |
|  | FEV1/FVC post (%) | 71 | 44 | 88 | 87 | 44 |
|  | CAT test score total | 4 | 12 | 6 | 9 | 11 |
|  | **Medication** |  |  |  |  |  |
|  | SABA/SAMA (yes/no) | no | no | no | no | no |
|  | LABA/LAMA (yes/no) | yes | yes | no | no | no |
|  | ICS (yes/no) | yes | yes | no | no | no |
|  | OCS (yes/no) | no | no | no | no | no |

BMI (body mass index); COPD (Chronic Obstructive Pulmonary Disease); CS (Current Smoker); ES (Ex-Smoker); NS (Never Smoker); FEV1 pre/post (forced expiratory volume 1 second pre/post-bronchodilator); FVC pre (forced vital capacity pre-bronchodilator); SABA/SAMA (short-acting beta agonist/short-acting muscarin antagonist); LABA/LAMA (long-acting beta agonist/long-acting muscarin antagonist); ICS (inhalation corticosteroids); OCS (oral corticosteroids).

**Supplementary Table 2. Antibodies used for immunohistochemistry and flow cytometry.**

|  |  | **Company** | **Clone** | **Dilution** |
| --- | --- | --- | --- | --- |
|  | **Immunohistochemistry** |  |  |  |
|  | Rat anti-human IL-10 | Thermo Fisher Scientific | JES3-12G8 | 1/10 |
|  | Rat IgG2a κ isotype control | BD Biosciences | R35-95 | 1/5 |
|  | Donkey anti-rat AF488 | Thermo Fisher Scientific | NA | 1/250 |
|  | Mouse anti-human CD20 | Abcam | L26 | 1/50 |
|  | Mouse IgG2a κ isotype control | BD Biosciences | C1.18.4 | 1/4.2 |
|  | Donkey anti-mouse AF647 | Thermo Fisher Scientific | NA | 1/250 |
|  | DAPI | Thermo Fisher Scientific | NA | 1/460 |
|  | **Flow cytometry** |  |  |  |
|  | Unconjugated Human IgG | Sigma Aldrich | NA | 1/100 |
|  | Fixable Viability Stain 575 V | BD Biosciences | NA | 1/1000 |
|  | CD20-AF700 | Thermo Fisher Scientific | 2H7 | 1/20 |
|  | CD19-APC-eFluor780 | Thermo Fisher Scientific | HIB19 | 1/50 |
|  | IgD-FITC | Biolegend | IA62 | 1/100 |
|  | CD27-PE | BD Biosciences | M-T271 | 1/20 |
|  | IgM-BV510 | BD Biosciences | MHM-88 | 1/50 |
|  | IL10-PE-CF594 | BD Biosciences | JES3-19F1 | 1/20 |
|  | IL6-BV421 | BD Biosciences | MQ2-13A5 | 1/20 |

**Supplementary Table 3. Characteristics of subjects enrolled in HIF-1α and IRF4 quantification upon air- and CSE exposure (n=19).**

|  |  | **Subjects** |
| --- | --- | --- |
|  |  | N=19 |
|  | **Age (years)** | 63 (8) |
|  | **Sex (female/male)** | 10/9 |
|  | **BMI** | 29 (11) |
|  | **Smoking** |  |
|  | Smoking status (CS/ES/NS) | 3/10/6 |
|  | Packyears | 25 (20) |
|  | **Medication** |  |
|  | SABA/SAMA (yes/no) | 3/16 |
|  | LABA/LAMA (yes/no) | 7/12 |
|  | ICS (yes/no) | 4/15 |
|  | OCS (yes/no) | 0/19 |

BMI (body mass index); CS (Current Smoker); ES (Ex-Smoker); NS (Never Smoker); SABA/SAMA (short-acting beta agonist/short-acting muscarin antagonist); LABA/LAMA (long-acting beta agonist/long-acting muscarin antagonist); ICS (inhalation corticosteroids); OCS (oral corticosteroids). Data are represented as mean (standard deviation).

**FIGURE LEGENDS**

**Supplementary Figure 1. Gating strategy to identify B cell subsets**

Representative example of the gating strategy used to define the following subsets: A) lymphocytes within single cell populations, B) alive CD20+ B cells within lymphocytes, C) CD27 and IgD expression within the total B cells population, D) naïve B cells (CD27-IgD+IgM+), E) natural effector cells (CD27+IgD+IgM+), and F) activated memory B cells (CD27+IgD-IgM+) and class-switched memory B cells (CD27+IgD-IgM-).

**Supplementary Figure 2. Gating strategy to determine IL-10 and IL-6 expression**

Representative example of the gating strategy used to determine IL-6+ and IL-10+ cells within total B cells and the different B cell subsets as defined in Supplementary Figure 2.

**Supplementary Figure 3. IHC stain for IL-10 and CD20 with isotype control**

Representative image of IL-10 and CD20 immunohistochemical staining showing A) positive signal for IL-10 (green), CD20 (red), and nuclei (grey), B) negative signal when the tissue section was stained with isotype controls. All images were taken at a 630x magnification. Scale bar length is 10 µm.

**Supplementary Figure 4. Ratios of IL6+ B-effs to IL10+ B-regs in peripheral blood from healthy controls, smokers, and COPD patients determined by flow cytometry.**

Ratios of IL6+ B-effs to IL10+ B-regs within total B lymphocytes (A) and B cell subsets (B-E) are shown.

**Supplementary Figure 5. Capacity of magnetically sorted B cells to produce IL-10 upon cigarette-exposure with BAFF inhibition.**

The capacity of MACS sorted B cells to produce IL-10 upon cigarette smoke exposure with and without the addition of BAFF-Fc-Chimera (inhibition of BAFF) was determined by flow cytometry and ELISA: A) Percentages of IL10+ cells within unstimulated cells, stimulated cells in medium, 5% CSE, and 10% CSE without the addition of BAFF-Fc-Chimera (white) and with the addition of BAFF-Fc-Chimera (grey) determined by flow cytometry, B) Levels of IL-10 in supernatant of stimulated cells in medium, 5% CSE, and 10% CSE without the addition of BAFF-Fc-Chimera (white) and with the addition of BAFF-Fc-Chimera (grey) determined by ELISA.
