# Supplementary figures and images for: IL-10 producing regulatory B cells are decreased in blood from smokers and COPD patients
Source: Respir Res. 2022 Oct 17;23:287. doi: 10.1186/s12931-022-02208-1 (PMC9578234; doi:10.1186/s12931-022-02208-1)

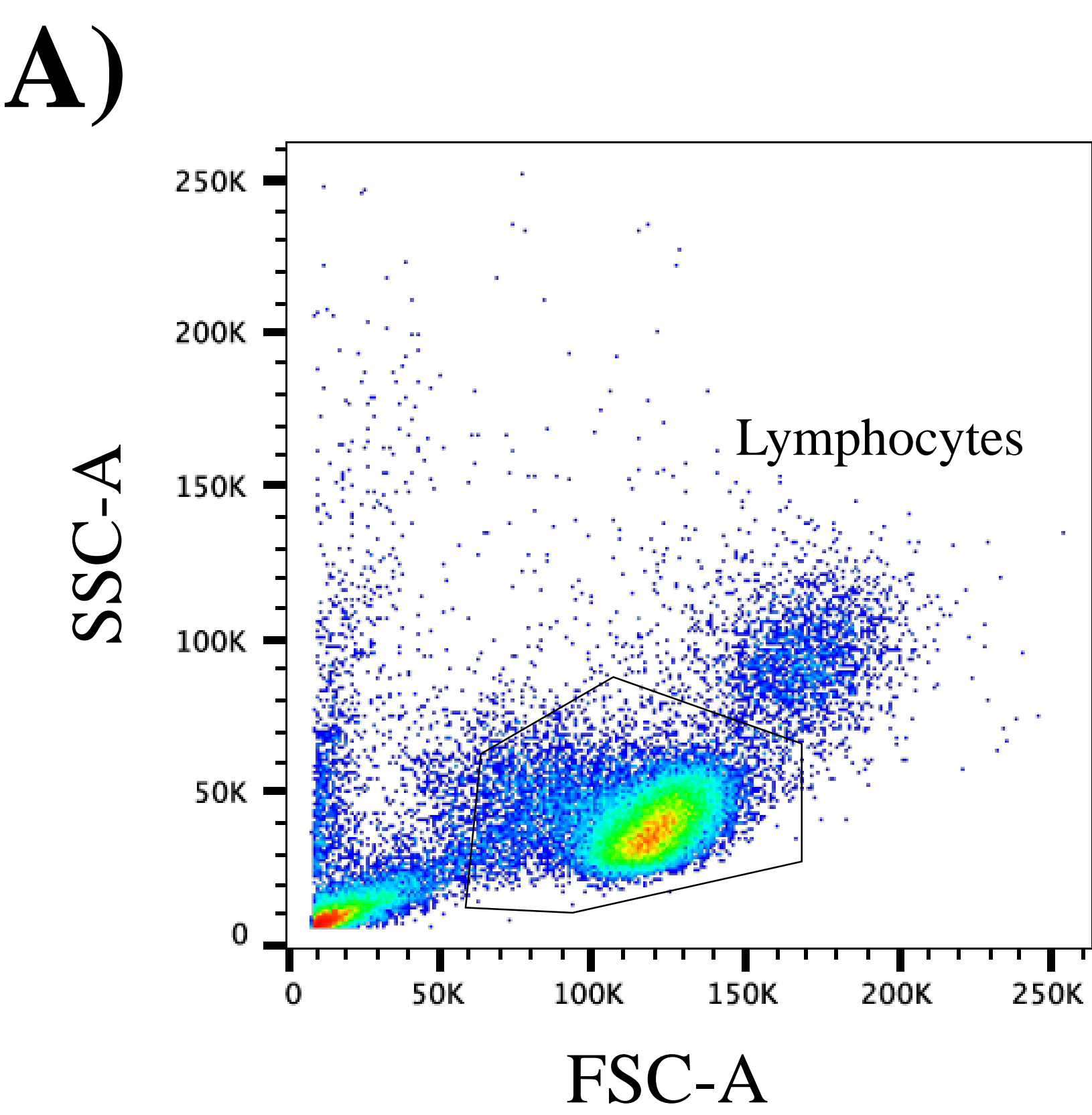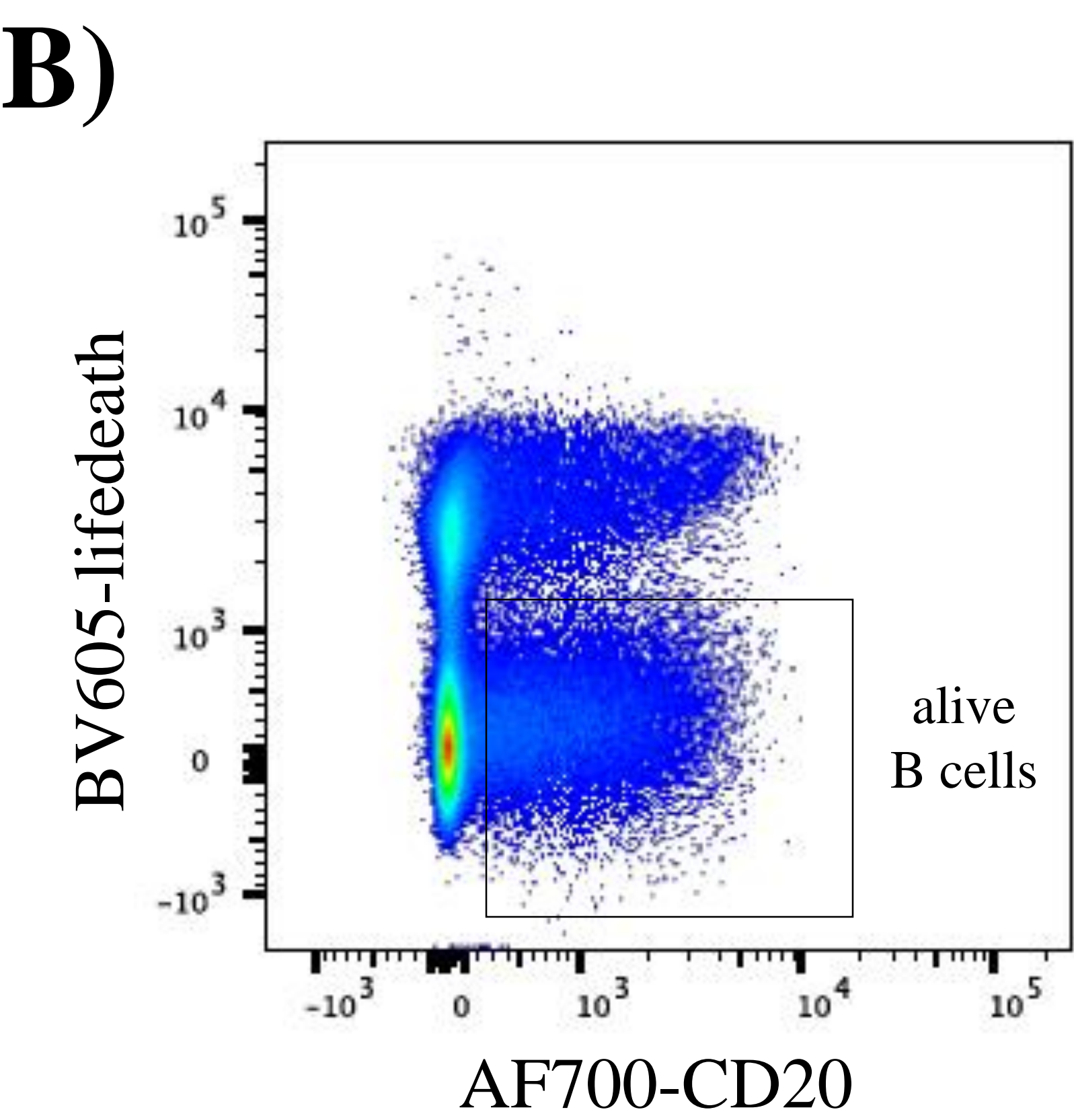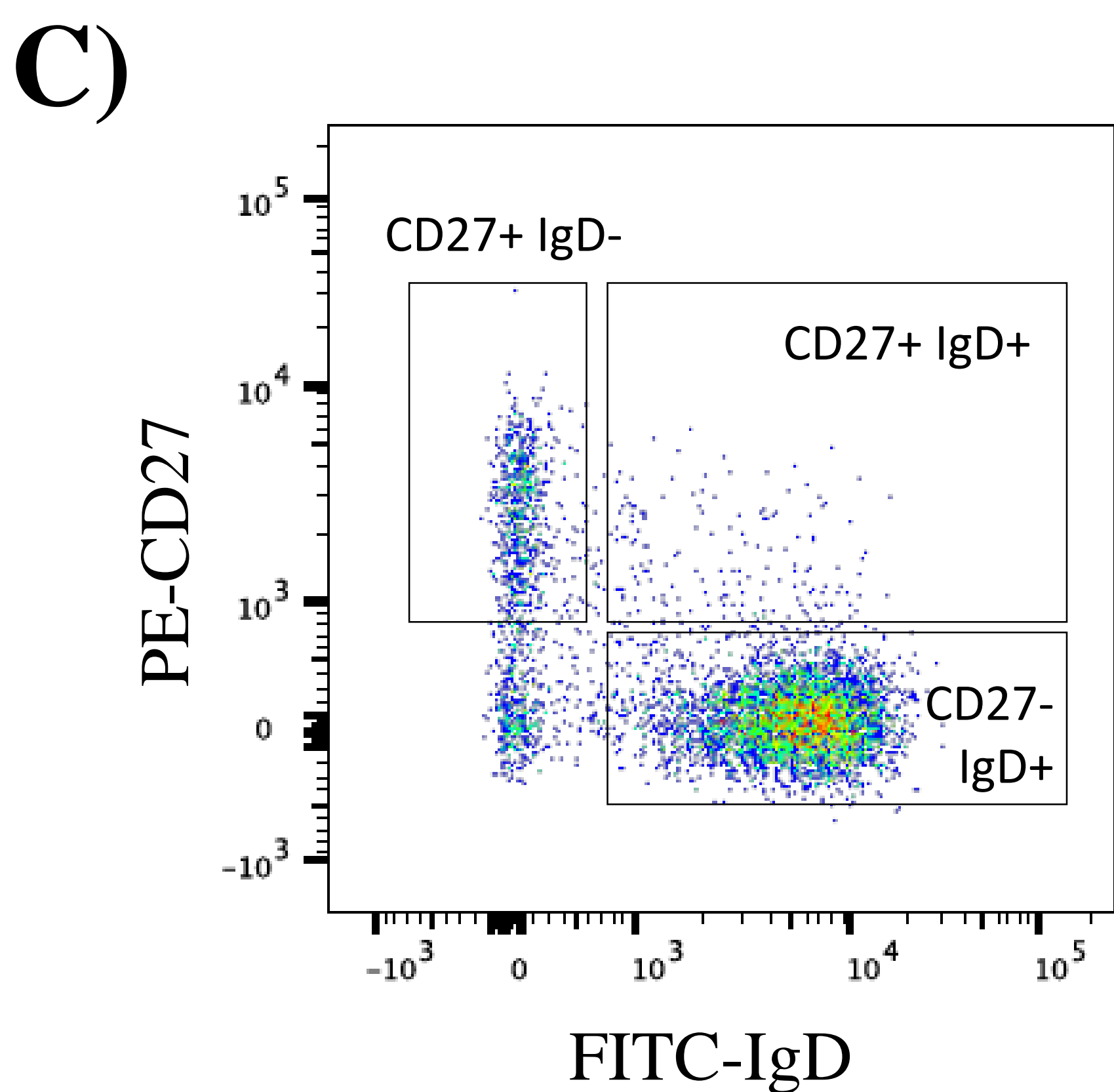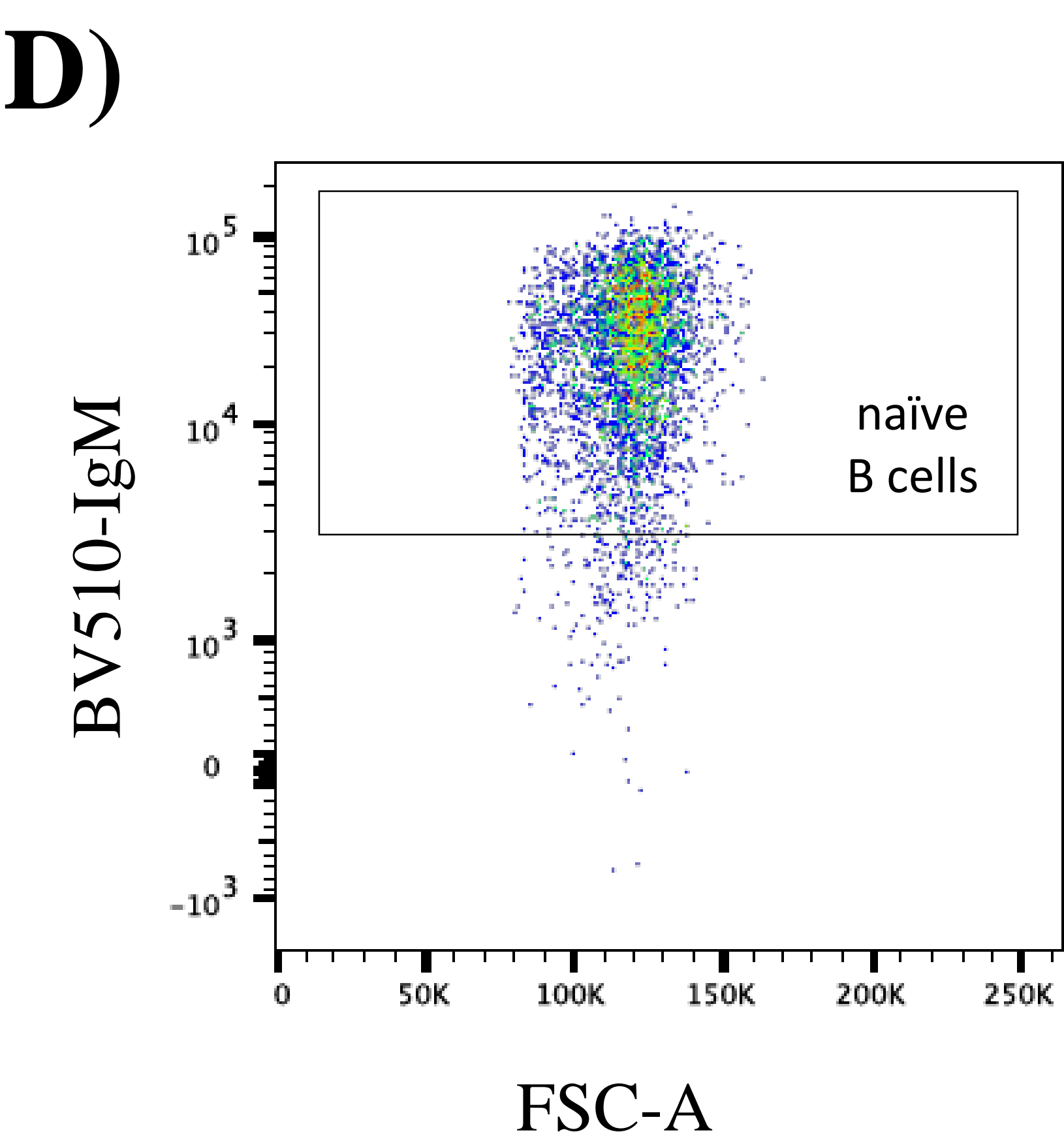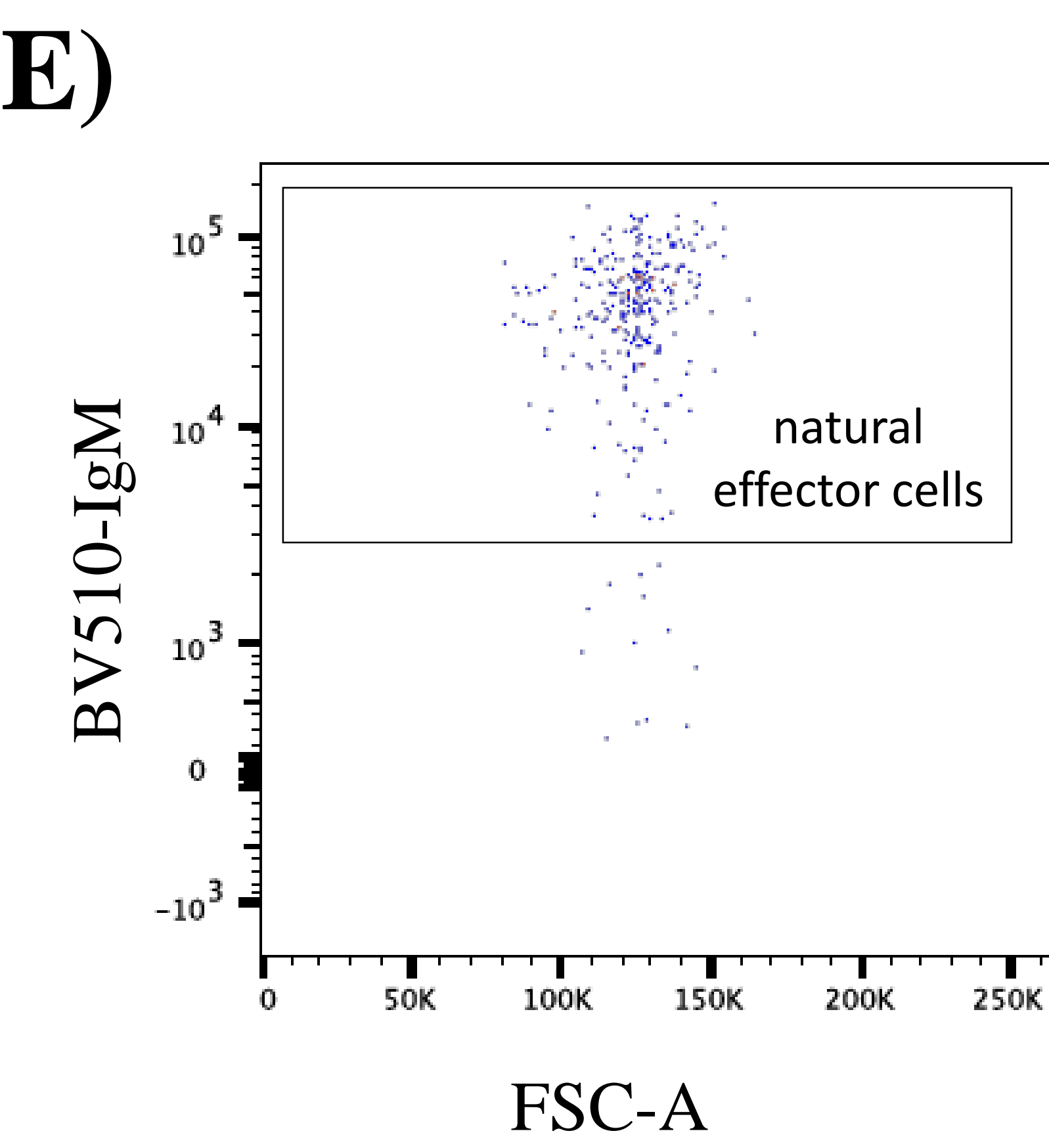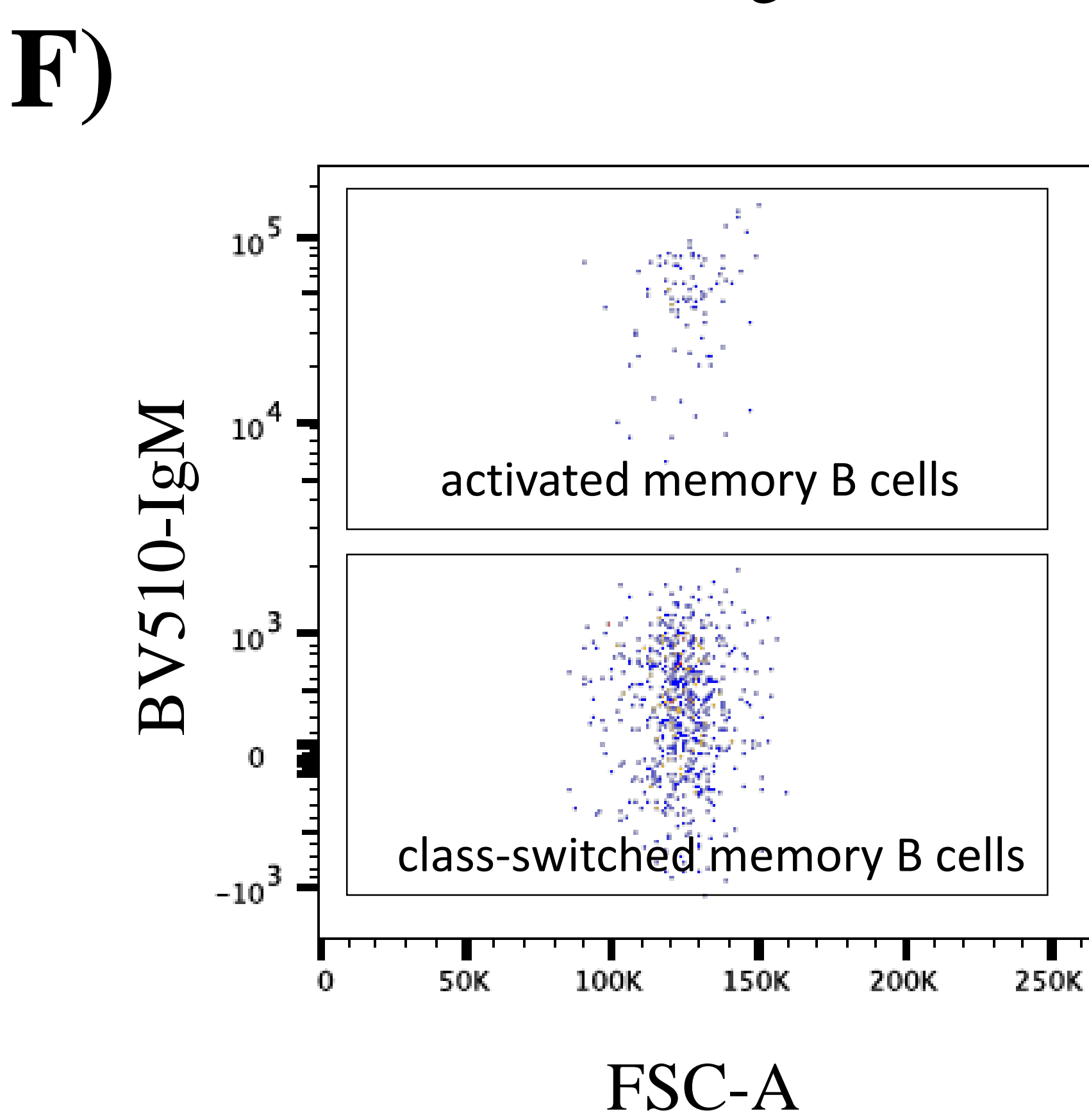

**Supplementary Figure 1.**

Supplement: Supplementary file 2 — Additional file 2. Supplementary Figure 1. Gating strategy to identify B cell subsets. Representative example of the gating strategy used to define the following subsets: A) lymphocytes within single cell populations, B) alive CD20+ B cells within lymphocytes, C) CD27 and IgD expression within the total B cells population, D) naïve B cells (CD27-IgD+IgM+), E) natural effector cells (CD27+IgD+IgM+), and F) activated memory B cells (CD27+IgD-IgM+) and class-switched memory B cells (CD27+IgD-IgM-). [file 12931_2022_2208_MOESM2_ESM.pdf]

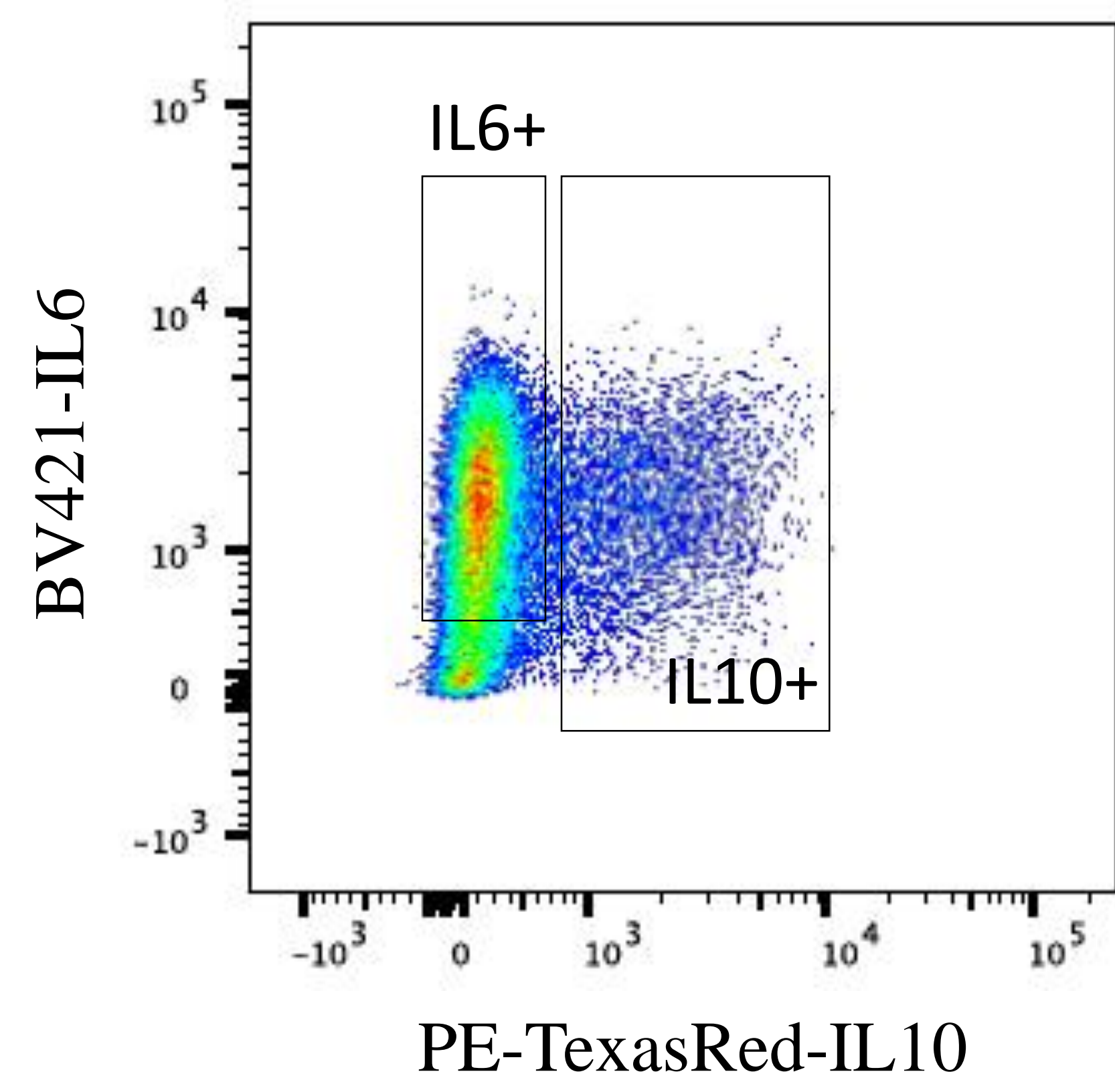

**Supplementary Figure 2.**

Supplement: Supplementary file 3 — Additional file 3. Supplementary Figure 2. Gating strategy to determine IL-10 and IL-6 expression. Representative example of the gating strategy used to determine IL-6+ and IL-10+ cells within total B cells and the different B cell subsets as defined in Supplementary Figure 2. [file 12931_2022_2208_MOESM3_ESM.pdf]

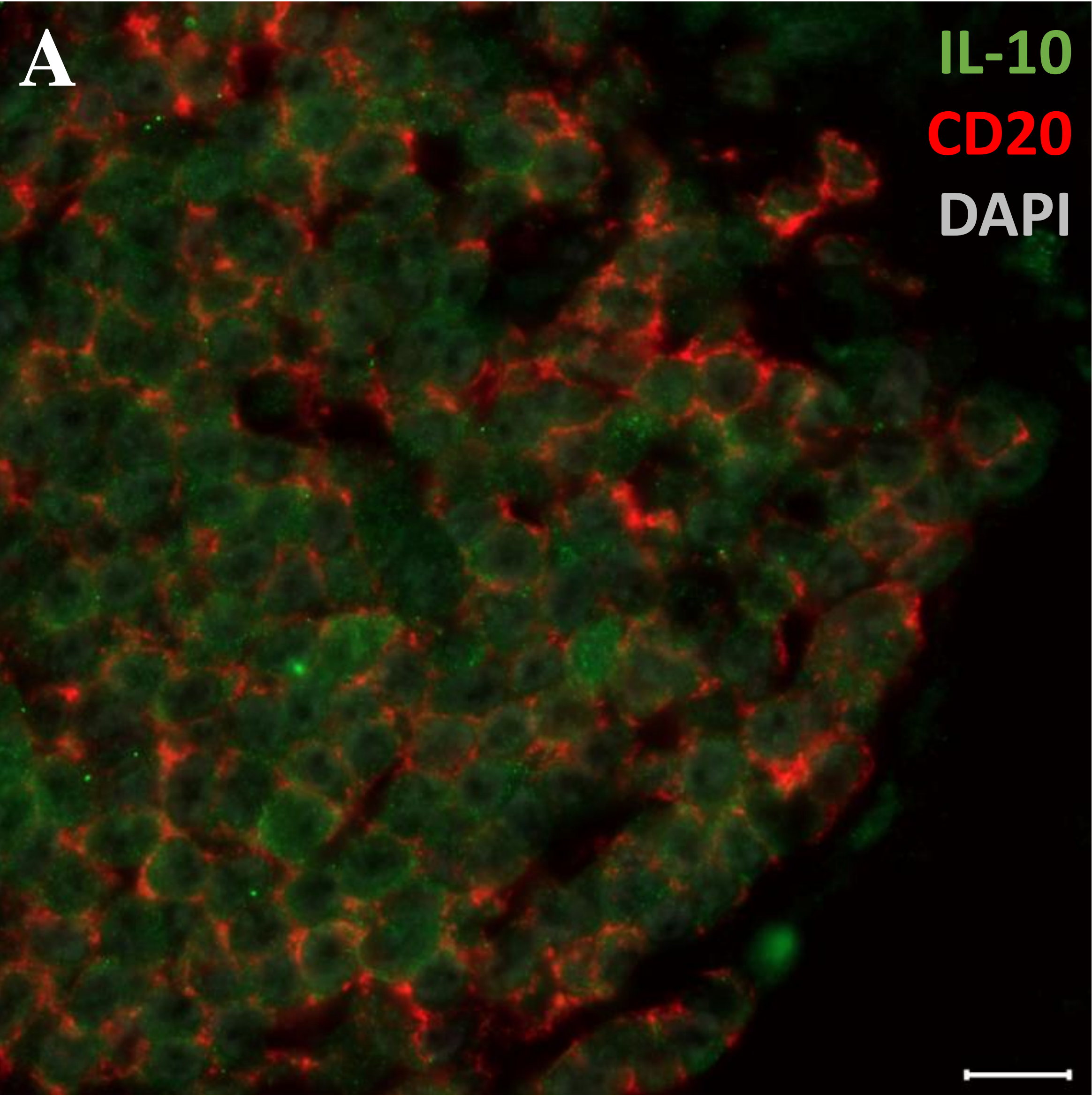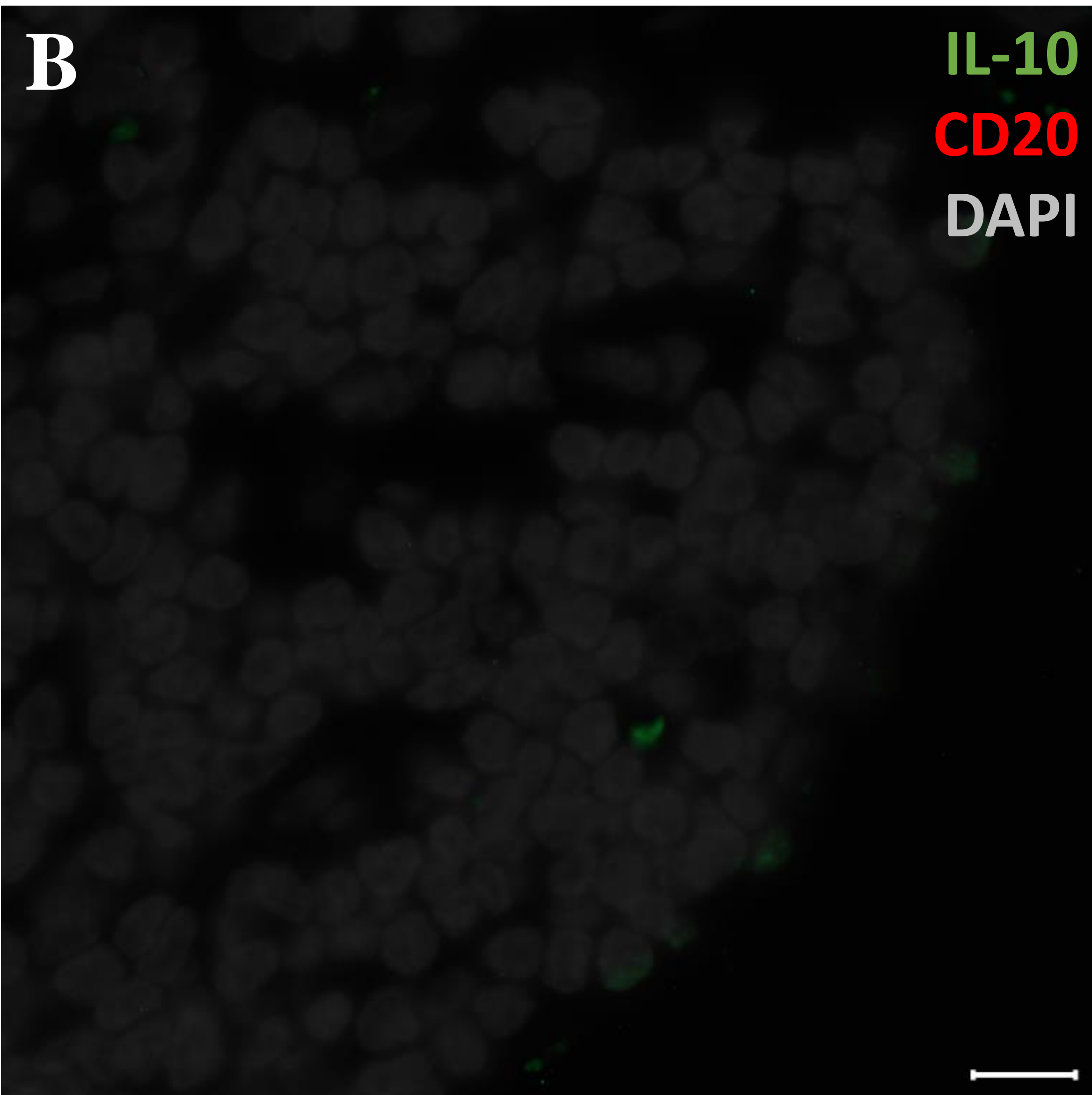

Supplementary Figure 3.

Supplement: Supplementary file 4 — Additional file 4. Supplementary Figure 3. IHC stain for IL-10 and CD20 with isotype control. Representative image of IL-10 and CD20 immunohistochemical staining showing A) positive signal for IL-10 (green), CD20 (red), and nuclei (grey), B) negative signal when the tissue section was stained with isotype controls. All images were taken at a 630x magnification. Scale bar length is 10 μm. [file 12931_2022_2208_MOESM4_ESM.pdf]

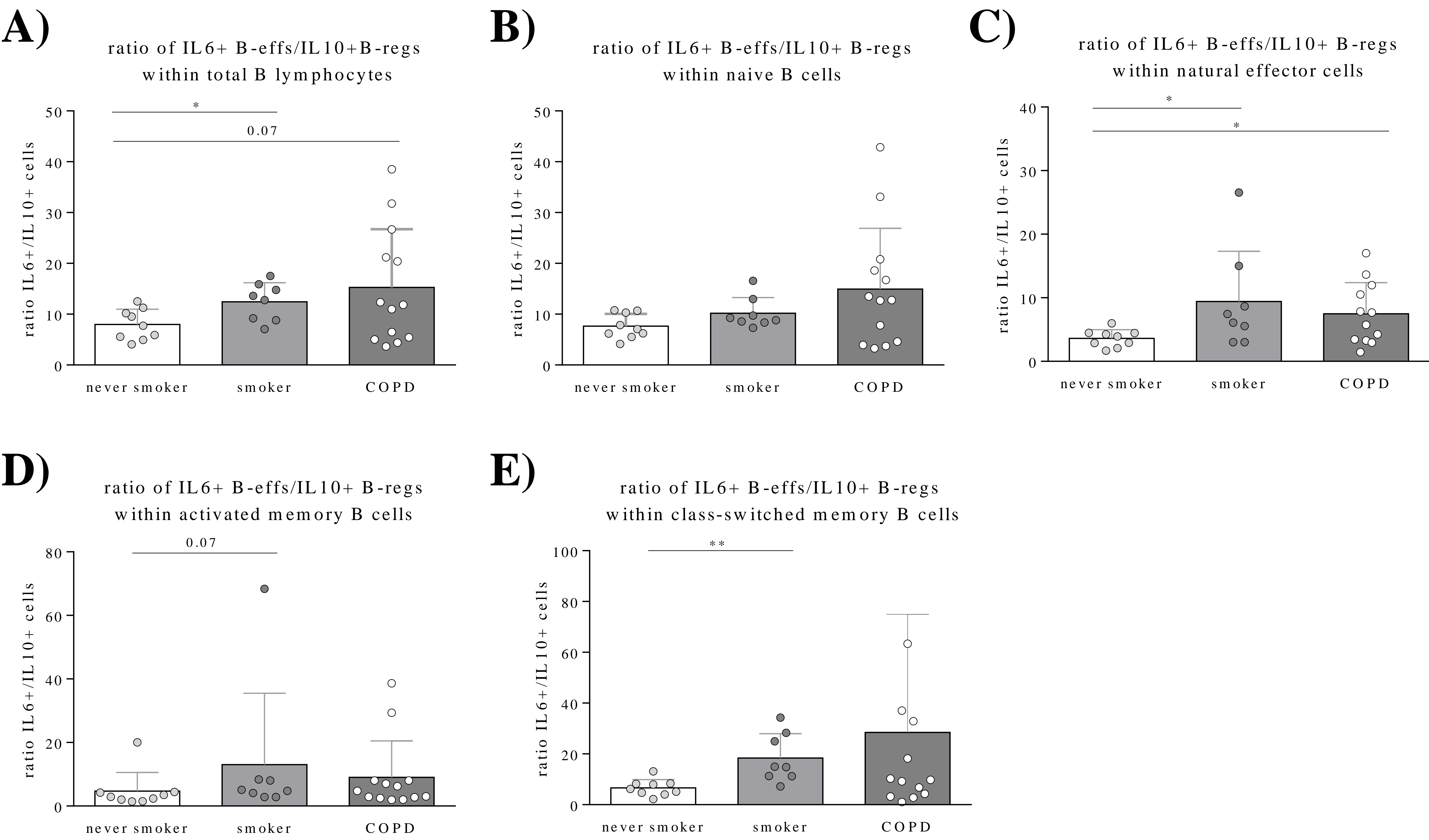

**Supplementary Figure 4.**

Supplement: Supplementary file 5 — Additional file 5. Supplementary Figure 4. Ratios of IL6+ B-effs to IL10+ B-regs in peripheral blood from healthy controls, smokers, and COPD patients determined by flow cytometry. Ratios of IL6+ B-effs to IL10+ B-regs within total B lymphocytes (A) and B cell subsets (B-E) are shown. [file 12931_2022_2208_MOESM5_ESM.pdf]

A)

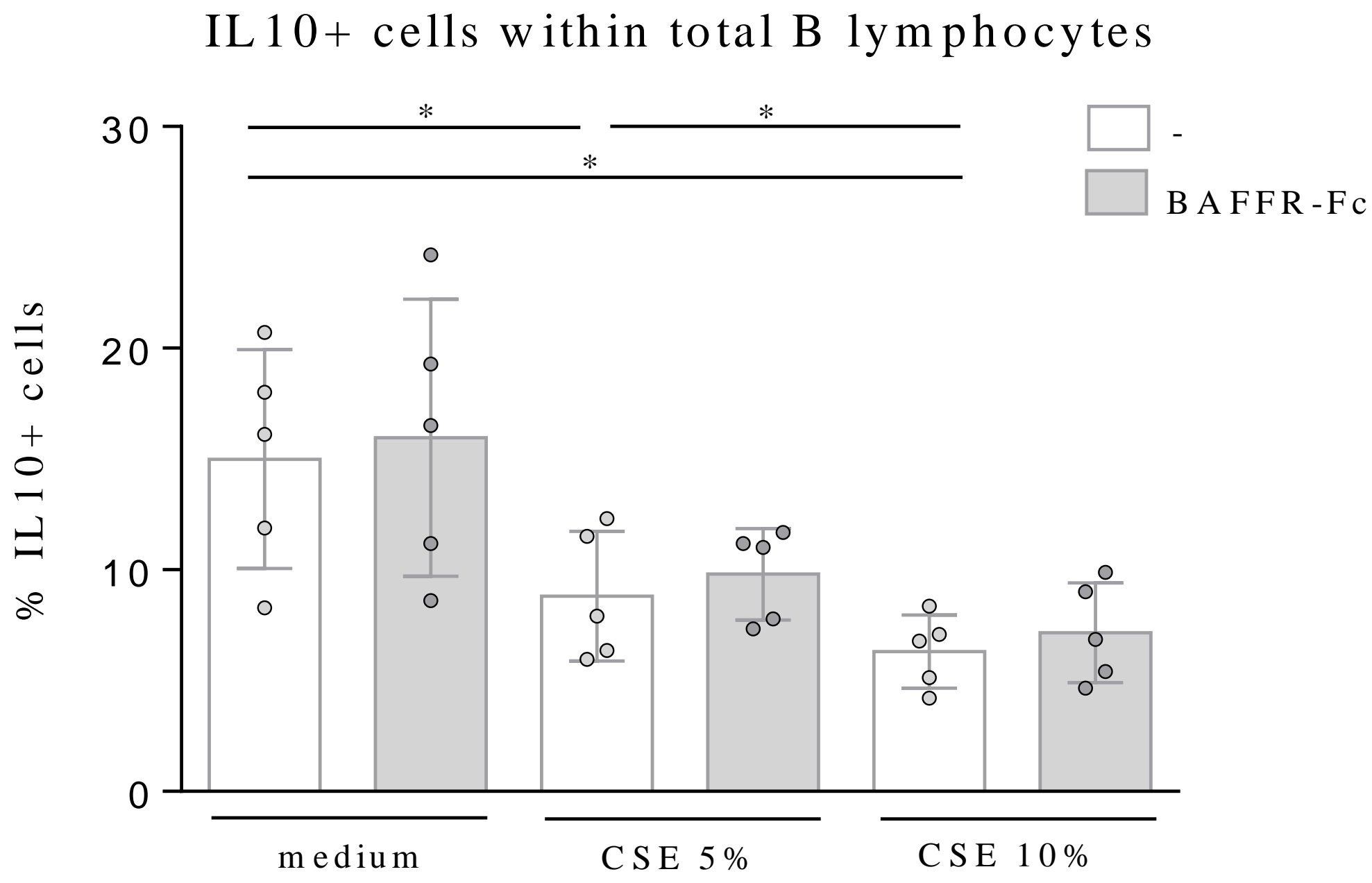

B)

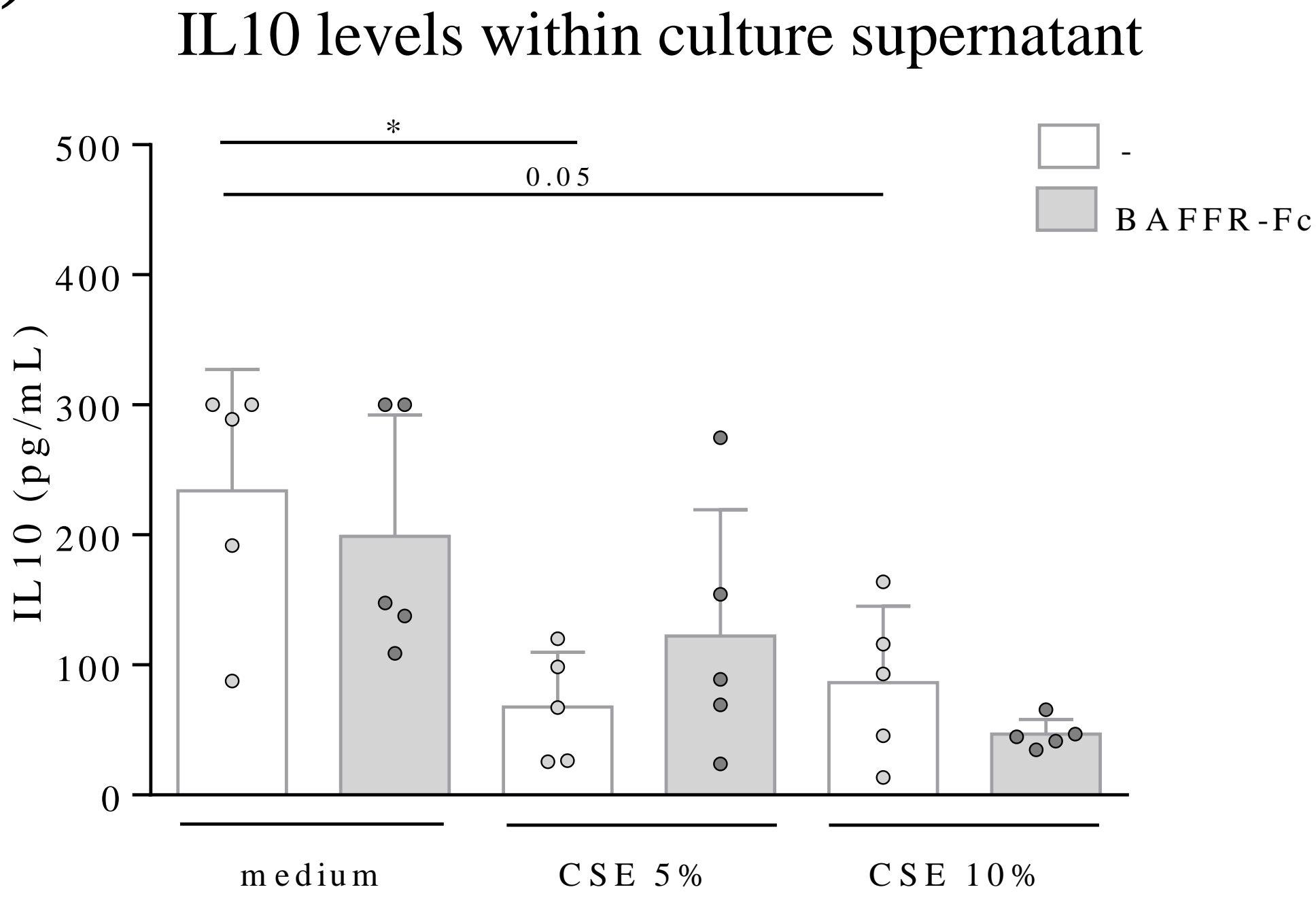

Supplementary Figure 5.

Supplement: Supplementary file 6 — Additional file 6. Supplementary Figure 5. Capacity of magnetically sorted B cells to produce IL-10 upon cigarette-exposure with BAFF inhibition. The capacity of MACS sorted B cells to produce IL-10 upon cigarette smoke exposure with and without the addition of BAFF-Fc-Chimera (inhibition of BAFF) was determined by flow cytometry and ELISA: A) Percentages of IL10+ cells within unstimulated cells, stimulated cells in medium, 5% CSE, and 10% CSE without the addition of BAFF-Fc-Chimera (white) and with the addition of BAFF-Fc-Chimera (grey) determined by flow cytometry, B) Levels of IL-10 in supernatant of stimulated cells in medium, 5% CSE, and 10% CSE without the addition of BAFF-Fc-Chimera (white) and with the addition of BAFF-Fc-Chimera (grey) determined by ELISA. [file 12931_2022_2208_MOESM6_ESM.pdf]
